# Incidences and variations of hospital acquired venous thromboembolism in Australian hospitals: a population-based study

**DOI:** 10.1186/s12913-016-1766-y

**Published:** 2016-09-22

**Authors:** Hassan Assareh, Jack Chen, Lixin Ou, Ken Hillman, Arthas Flabouris

**Affiliations:** 1 Epidemiology and Health Analytics, Western Sydney Local Health Districts, Gungurra Building 68, Cumberland Hospital, 5 Fleet Street, North Parramatta, 2151 NSW Australia; 2 Simpson Centre for Health Services Research-South Western Sydney Clinical School Faculty of Medicine, University of New South Wales, and Ingham Institute, Sydney, Australia; 3 Intensive Care Unit, Royal Adelaide Hospital and School of Medicine, Faculty of Health Sciences, University of Adelaide, Adelaide, South Australia Australia

**Keywords:** Hospital acquired complication, Patient safety, Quality improvement, Venous thromboembolism

## Abstract

**Background:**

Data on hospital-acquired venous thromboembolism (HA-VTE) incidence, case fatality rate and variation amongst patient groups and health providers is lacking. We aim to explore HA-VTE incidences, associated mortality, trends and variations across all acute hospitals in New South Wales (NSW)-Australia.

**Methods:**

A population-based study using all admitted patients (aged 18–90 with a length of stay of at least two days and not transferred to another acute care facility) in 104 NSW acute public and private hospitals during 2002–2009. Poisson mixed models were used to derive adjusted rate ratios (IRR) in presence of patient and hospital characteristics.

**Results:**

Amongst, 3,331,677 patients, the incidence of HA-VTE was 11.45 per 1000 patients and one in ten who developed HA-VTE died in hospital. HA-VTE incidence, initially rose, but subsequently declined, whereas case fatality rate consistently declined by 22 % over the study period. Surgical patients were 128 % (IRR = 2.28, 95 % CI: 2.19–2.38) more likely to develop HA-VTE, but had similar case fatality rates compared to medical patients. Private hospitals, in comparison to public hospitals had a higher incidence of HA-VTE (IRR = 1.76; 95 % CI: 1.42–2.18) for medical patients. However, they had a similar incidence (IRR = 0.91; 95 % CI: 0.75–1.11), but a lower mortality (IRR = 0.59; 95 % CI: 0.47–0.75) amongst surgical patients. Smaller public hospitals had a lower HA-VTE incidence rate compared to larger hospitals (IRR < 0.68) but a higher case fatality rate (IRR > 1.71). Hospitals with a lower reported HA-VTE incidence tended to have a higher HA-VTE case fatality rate.

**Conclusion:**

Despite the decline in HA-VTE incidence and case fatality, there were large variations in incidents between medical and surgical patients, public and private hospitals, and different hospital groups. The causes of such differences warrant further investigation and may provide potential for targeted interventions and quality improvement initiatives.

**Electronic supplementary material:**

The online version of this article (doi:10.1186/s12913-016-1766-y) contains supplementary material, which is available to authorized users.

## Background

Venous thromboembolism (VTE), comprising both deep venous thrombosis (DVT) and pulmonary embolism (PE) are potentially preventable and treatable medical conditions that can contribute to patient morbidity and mortality [1, 2]. VTE accounts for almost 10 % of all hospital deaths [3], and over half of VTE incidents are hospital acquire (HA-VTE) [4–6]. Appropriate intervention (e.g. pharmacological and mechanical prophylaxis), can significantly reduce the incidence of VTE by 70 % for both medical and surgical patients [7–9]. Evidence-based VTE prevention and treatment guidelines [1, 10, 11] have been developed so as to reduce VTE occurrence and have been adopted for both accreditation and benchmarking [12, 13].

In the U.S the incidence, and fatality rates, of HA-VTE following surgery have decreased by 50 and 30 % respectively [14–16]. Effective implementation of VTE prevention and treatment protocols may have contributed to the decreased rates in the U.S. hospitals [17]. Substantial variation in trends and rates amongst similar hospitals [14], after adjustment for case mix and surgery types [18, 19], may reflect variation in compliance with VTE prevention strategies and the potential for further improvement [20]. In contrast, there has been a 30 % increase in post-operative VTE incidents in Australian hospitals [21].

There have been few studies of the HA-VTE incidence and subsequnt mortality in Australia [21–24]. In this study, we adopted a validated measure of HA-VTE to explore trends in the rates of HA-VTE, and associated mortality amongst admitted patients to all acute public and private hospitals in New South Wales (NSW), Australia between 2002 and 2009. Patients’ and hospitals’ contributing factors to the HA-VTE trends and variations were also examined.

## Methods

### Data source and study population

We used records from the NSW Admitted Patient Data Collection (APDC) database. It includes all admitted patients in NSW public and private, acute, sub-acute and non-acute facilities, and is used for health services planning, funding and research. Reporting to APDC is mandatory at all hospitals. The APDC includes information on patient demographics, medical conditions and procedures, hospital characteristics, and separations (discharges, transfers and deaths) from all public and private healthcare facilities in NSW. Each episode of care in the APDC is assigned with up to 55 codes for morbidities (principal diagnosis and comorbidities) based on the International Statistical Classification of Diseases and Related Health Problems, Tenth Revision, Australian Modification (ICD-10-AM) 4th edition [25]. The codes are assigned by trained and qualified clinical coders based on clinical notes and according to standards [26]. The quality of data was found reasonable, but varied across data elements and hospitals [27–29].

In NSW public hospitals accommodated 62 % of all patients’ admissions, with a greater proportion of overnight stays (70 %), but a similar same-day stays (51 %) compared to private hospitals. Over 80 % of funding for public and private hospitals were provided by government and health insurers respectively [30]. All admissions, between 1st January 2002 to 31st December 2009, from 104 of 497 NSW acute public and private hospitals (11,408,808, admissions; 71 %) were included. We excluded community, non-acute, psychiatric and rehabilitation facilities, nursing home and hospices, and the two children’s hospitals.

Included were patients who had a hospital length of stay (LOS) of at least two days, aged between 18 and 90 years (inclusive), and were not transferred to another acute care facility (4,089,144 episodes (35.8 %)).

### HA-VTE identification and covariates

Based on ICD-10-AM, we considered the codes I26 (.0) and I26.9 for PE and I80.1, I80.2, I80.3, I80.8, I80.9, I82.8, and I82.9 for DVT cases - a total of nine VTE diagnosis codes. VTE codes were chosen according to well-established VTE related measures within a quality and safety context [12, 31, 32] and existing published coding procedures [19, 22]. Cases with no VTE code as the principal diagnosis were identified as patients at risk and those who had at least one VTE code in secondary diagnoses were identified as HA-VTE cases. It resulted in exclusion of patients with VTE as principal diagnoses from both numerator and denominator. Obstetric patients, identified by major diagnosis category using Australian Refined Diagnosis Related Groups (AR-DRG) [25], and those surgical patients who only had a procedure for interruption of vena cava (ICD codes: 348000, 3533000 and 3533001) were excluded from the study population as suggested within a patient safety context in Australia [32] and elsewhere [31, 33]. The discharge status (alive or deceased) was used to derive the case fatality associated with a HA-VTE diagnosis. HA-VTE cases and related deaths were respectively presented as incidence rate (per 1000 admissions) and case fatility.

For all admissions, two sets of patient- and hospital-related covariates were considered. Patient demographic variables included age, gender, country of birth, marital status, patient socio-economic status, principal diagnostic disease groups (the ten most common groups based on Elixhauser comorbidities [34]), and length of stay within the study population. We utilised a postcode-level advantage and disadvantage index of Socio-Economic Indices for Areas (SEIFA) with the lower values indicating more disadvantaged areas [35]. SEIFA scores were categorised into four classes (1st quartile = most disadvantaged areas and 4th quartile = most advantaged areas). The disease groups were identified using principal diagnostic codes (ICD-10-AM) at admissions through the methodology developed by Quan et al. [34]. Admissions with any surgical codes recored as the principal procedure were categorised as surgical admissions and those with no procedural data were categorised as medical admissions. Using relevant procedure codes from ICD-10-AM (Additional file 1), we defined six major surgical procedures including coronary-artery bypass graft (CABG), abdominal aortic aneurysm (AAA) repair, total hip replacement, total knee replacement, cholecystectomy, and other surgical procedures.

Hospital covariates included the local health district (metropolitan, rural and regional NSW), the hospital type (public and private), and associated peer groups. Public hospital peer groups included “A1”: principal referral group, usually teaching hospitals; “A3”: ungrouped acute; “B”: major metropolitan and non-metropolitan; “C1”: district group 1; and, “C2”: district group 2. Private hospital peer groups included “21”: major; and “22”: district group hospitals. Hospital peer groups contained similar type and sized hospitals, ranging from those treating more than 25,000 acute case-mix weighted separations per annum in principal referral groups through to treating 2000+ (but less than 5000) acute case-mix weighted separations per annum in district group [36].

### Statistical analysis

We employed Poisson mixed models to evaluate adjusted incidence and case fatality rates and rate ratios for both study outcomes after including all patients and hospital-related characteristics. A random intercept term was utilised to incorporate any clustering effect at hospital-level. Stratified models were constructed to derive estimates for specific admission types (medical and surgical) and hospital peer groups (public and private) separately. To investigate the temporal pattern of the outcomes, calendar years were entered into the model as indicator variables, with 2002 as the reference year. A model with the year as a continuous variable was also examined for linear trends. We derived specific trends for hospital types, admission type, peer group and surgery type using separate models. Adjusted rates for specific years were derived by multiplying yearly-adjusted rate ratios to the crude rates observed in the reference year.

We did not include either the Elixhauser or the Charlson Index comorbidities in the models due to an approximately unexpected 50 % drop (see Additional file 2) observed in the indices among our study population in recent years [21], and also reported geographical variations and biases in the coding that may lead to misleading results [37, 38]. For example, it was reported that there is an increased under-recording of diabetes conditions in APDC datasets between 2008 and 2012 [39].

To study the variation of outcomes across hospitals within each hospital group, hospital-specific random intercept components were extracted from stratified Poisson mixed models constructed for each hospital group, then ranked and categorised into five classes at 20 % incremental quintiles. To obtain adjusted differences between those with the highest and those with the lowest HA-VTE and HA-VTE case fatality rates, the adjusted classes were entered into a Poisson model including patient characteristics covariates. We then used Pearson correlation to assess the association of hospital performances between HA-VTE and post HA-VTE deaths, based on the hospital-specific random intercepts. All analyses were performed in R package version 3.0.0 [40] and Stata™ 11.0 [41].

## Result

### The distributions of study outcomes by patient and hospital characteristics

Of the 3,331,677 admissions during 2002–2009 with a median LOS of five days (1^st^ and 3^rd^ quartiles: 3, 9 days), 38,161 patients developed a HA-VTE, resulting in an incidence rate of 11.4 per 1000 patients (median LOS = 12, quartiles: 7–22 days; Table 1). Among them, 3716 (9.7 %) died in hospitals (median LOS = 13, quartiles: 7–25 days). Patients who underwent surgery (elective or non-elective, 83 % of all included patients) accounted for 93.3 % of HA-VTE cases and were 2.3 times more likely to develop HA-VTE compared to medical patients, but had a similar mortality rate. Compared to females, male patients were less likely to develop a HA-VTE (IRR = 0.93) but more likely to die (IRR = 1.25). HA-VTE incidence and associated case fatality were higher among elderly. Patients who stayed longer in hospitals had higher HA-VTE incidences but lower fatalities.

**Table 1:** Study population, incidence rates and adjusted rate ratios of patients who developed HA-VTE and associated case fatality

Characteristics	All Patients		HA-VTE						HA-VTE case fatality					
	N	(%)	n	(%)	IR	IRR	(95 % CI)		n	(%)	%	IRR	(95 % CI)	
Admission type^a^														
Medical	573,042	(17.20 %)	2,565	(6.72 %)	4.5	1.00			245	(6.59 %)	9.6 %	1.00		
Surgical	2,758,635	(82.80 %)	35,596	(93.28 %)	12.9	2.28	(2.19–2.38)	^d^	3,471	(93.41 %)	9.8 %	1.05	(0.91–1.20)	
Sex														
Female	1,693,109	(50.82 %)	19,805	(51.90 %)	11.7	1.00			1,730	(46.56 %)	8.7 %	1.00		
Male	1,638,568	(49.18 %)	18,356	(48.10 %)	11.2	0.93	(0.91–0.95)	^d^	1,986	(53.44 %)	10.8 %	1.25	(1.17–1.33)	^d^
Age														
> =75 years & <90	1,074,206	(32.24 %)	15,350	(40.22 %)	14.3	1.00			1,832	(49.30 %)	11.9 %	1.00		
> =18 years & <35 years	396,444	(11.90 %)	1,742	(4.56 %)	4.4	0.53	(0.50–0.56)	^d^	55	(1.48 %)	3.2 %	0.23	(0.17–0.30)	^d^
> =35 years & <55 years	707,274	(21.23 %)	5,612	(14.71 %)	7.9	0.83	(0.81–0.86)	^d^	354	(9.53 %)	6.3 %	0.47	(0.41–0.52)	^d^
> =55 years & <75 years	1,153,753	(34.63 %)	15,457	(40.50 %)	13.4	1.13	(1.10–1.15)	^d^	1,475	(39.69 %)	9.5 %	0.77	(0.72–0.83)	^d^
Marital status														
Married	1,803,208	(54.12 %)	20,814	(54.54 %)	11.5	1.00			2,089	(56.22 %)	10.0 %	1.00		
Single	1,372,496	(41.20 %)	15,730	(41.22 %)	11.5	0.90	(0.89–0.92)	^d^	1,447	(38.94 %)	9.2 %	0.91	(0.85–0.98)	^c^
Unknown	155,973	(4.68 %)	1,617	(4.24 %)	10.4	0.83	(0.79–0.88)	^d^	180	(4.84 %)	11.1 %	1.12	(0.96–1.31)	
Country of birth														
Australia and New Zealand	2,411,315	(72.38 %)	26,794	(70.21 %)	11.1	1.00			2,575	(69.29 %)	9.6 %	1.00		
UK, US & Canada	223,710	(6.71 %)	2,873	(7.53 %)	12.8	1.01	(0.97–1.05)		271	(7.29 %)	9.4 %	0.90	(0.79–1.02)	
Non-English Europe	317,973	(9.54 %)	4,546	(11.91 %)	14.3	0.98	(0.95–1.02)		493	(13.27 %)	10.8 %	0.98	(0.88–1.08)	
North Africa	61,595	(1.85 %)	487	(1.28 %)	7.9	0.67	(0.61–0.73)	^d^	47	(1.26 %)	9.7 %	1.09	(0.82–1.46)	
Asia	101,485	(3.05 %)	996	(2.61 %)	9.8	0.76	(0.71–0.81)	^d^	98	(2.64 %)	9.8 %	1.11	(0.90–1.36)	
Others	215,599	(6.47 %)	2,465	(6.46 %)	11.4	0.94	(0.90–0.98)	^d^	232	(6.24 %)	9.4 %	1.01	(0.88–1.16)	
Quartiles of SEIFA														
1st quartile (most disadvantaged)	913,712	(27.42 %)	9,482	(24.85 %)	10.4	1.00			939	(25.27 %)	9.9 %	1.00		
2nd quartile (disadvantaged)	862,580	(25.89 %)	8,843	(23.17 %)	10.3	1.00	(0.97–1.03)		900	(24.22 %)	10.2 %	1.05	(0.95–1.16)	
3rd quartile (advantaged)	827,343	(24.83 %)	9,701	(25.42 %)	11.7	0.98	(0.95–1.01)		1,011	(27.21 %)	10.4 %	1.12	(1.01–1.23)	^c^
4th quartile (most advantaged)	728,042	(21.85 %)	10,135	(26.56 %)	13.9	0.99	(0.95–1.03)		866	(23.30 %)	8.5 %	1.13	(1–1.27)	^c^
Length of stay														
2–4 days	1,495,477	(44.89 %)	4,803	(12.59 %)	3.2	1.00			567	(15.26 %)	11.8 %	1.00		
4–9 days	1,056,167	(31.70 %)	10,724	(28.10 %)	10.2	2.78	(2.69–2.88)	^d^	816	(21.96 %)	7.6 %	0.69	(0.62–0.76)	^d^
Over 10 days	780,033	(23.41 %)	22,634	(59.31 %)	29.0	7.91	(7.65–8.17)	^d^	2,333	(62.78 %)	10.3 %	0.82	(0.74–0.90)	^d^
Major principal diagnostic diseases^b^														
Cardiac arrhythmias	74,686	(2.24 %)	657	(1.72 %)	8.8	-			22	(0.59 %)	3.3 %	-		
Chronic pulmonary disease	129,030	(3.87 %)	853	(2.24 %)	6.6	-			92	(2.48 %)	10.8 %	-		
Coagulopathy	5,455	(0.16 %)	211	(0.55 %)	38.7	-			15	(0.40 %)	7.1 %	-		
Congestive heart failure	71,705	(2.15 %)	971	(2.54 %)	13.5	-			130	(3.50 %)	13.4 %	-		
Diabetes with chronic complication	41,956	(1.26 %)	464	(1.22 %)	11.1	-			58	(1.56 %)	12.5 %	-		
Malignancies	206,407	(6.20 %)	3,796	(9.95 %)	18.4	-			860	(23.14 %)	22.7 %	-		
Metastatic solid tumour	49,332	(1.48 %)	1,427	(3.74 %)	28.9	-			368	(9.90 %)	25.8 %	-		
Peripheral vascular disease	26,225	(0.79 %)	425	(1.11 %)	16.2	-			61	(1.64 %)	14.4 %	-		
Pulmonary circulation disorders	1,520	(0.05 %)	73	(0.19 %)	48.0	-			10	(0.27 %)	13.7 %	-		
Renal disease	8,736	(0.26 %)	103	(0.27 %)	11.8	-			14	(0.38 %)	13.6 %	-		
Rheumatic disease	9,811	(0.29 %)	176	(0.46 %)	17.9	-			9	(0.24 %)	5.1 %	-		
Hospital type														
Public	2,704,301	(81.17 %)	31,160	(81.65 %)	11.5	1.00			3,362	(90.47 %)	10.8 %	1.00		
Private	627,376	(18.83 %)	7,001	(18.35 %)	11.2	1.03	(0.85–1.25)		354	(9.53 %)	5.1 %	0.55	(0.43–0.71)	^d^
Peer hospital groups-Public														
Principal referral (A1)	1,315,038	(48.63 %)	19,134	(61.41 %)	14.6	1.00			2,069	(61.54 %)	10.8 %	1.00		
Ungrouped acute (A3)	73,569	(2.72 %)	765	(2.46 %)	10.4	0.67	(0.46–0.95)	^c^	132	(3.93 %)	17.3 %	2.71	(1.48–4.97)	^d^
Major metro- & non-metropolitan (B)	839,827	(31.06 %)	7,958	(25.54 %)	9.5	0.73	(0.60–0.89)	^d^	817	(24.30 %)	10.3 %	1.22	(0.91–1.64)	
District group 1 (C1)	245,005	(9.06 %)	1,933	(6.20 %)	7.9	0.64	(0.51–0.81)	^d^	198	(5.89 %)	10.2 %	1.75	(1.22–2.50)	^d^
District group 2 (C2)	230,862	(8.54 %)	1,370	(4.40 %)	5.9	0.48	(0.39–0.60)	^d^	146	(4.34 %)	10.7 %	2.06	(1.44–2.95)	^d^
Peer hospital groups-Private														
Major m	409,555	(65.28 %)	4,961	(70.86 %)	12.1	1.00			213	(60.17 %)	4.3 %	1.00		
District (22)	217,821	(34.72 %)	2,040	(29.14 %)	9.4	1.03	(0.64–1.64)		141	(39.83 %)	6.9 %	1.36	(0.79–2.34)	
Local health district														
Metropolitan	2,300,073	(69.04 %)	29,779	(78.04 %)	12.9	1.00			2,814	(75.73 %)	9.4 %	1.00		
Rural & Regional NSW	1,031,604	(30.96 %)	8,382	(21.96 %)	8.1	0.69	(0.59–0.81)	^d^	902	(24.27 %)	10.8 %	1.18	(0.95–1.45)	
Total	3,331,677		38,161		11.4				3,716		9.7 %			

111,493 (3.2 %) cases were excluded due to missing items

Incidence rates (IR) are crude and reported per 1000 patients

Incidence rate ratios (IRR) and related confident intervals (CI) were obtained using a Poisson mixed model and adjusted for patient and hospital characteristics

^a^IRRs were not adjusted for length of stay due to highly unbalanced distributions for medical versus surgical admissions

^b^No RR is reported since this characteristic has not been included in the Poisson mixed model

^c^Significant at 5 %; ^d^significant at 1 %

Patients who were born in Europe (except the UK), Asia and North Africa experienced a lower risk of post-operative HA-VTE but a similar risk of death. Higher socio-economic status (quartiles of SEIFA) was associated with a higher risk of post HA-VTE death, in particular for surgical patients. Surgical and medical patients admitted with pulmonary circulation or coagulopathy disorders had the highest HA-VTE incidences, followed by cancer patients who had the highest fatality rate. Patients who underwent total knee replacement, AAA repair and total hip replacement surgeries had a higher risk of HA-VTE. However, post HA-VTE mortality was lower amongst orthopaedic surgical patients, compared to other surgical patients (see Additional file 3).

For medical patients, HA-VTE incidence was significantly higher in private hospitals compared to public hospitals (IRR = 1.76), whereas for surgical patients it was similar (see Additional files 3 and 4). The risk of death in private hospitals was almost half of the risk in public hospitals, in particular for surgical patients.

Among public hospitals, patients from principal referral hospitals were more likely to acquire VTE during hospitalisation in comparison to patients from smaller hospitals. However, patients admitted to smaller hospitals, were at a higher risk of death. This pattern was almost consistent across medical and surgical patients (see Additional files 3 and 4). No differences in outcomes were observed between private hospital peer groups.

### The trends of HA-VTE incidence rate and HA-VTE case fatality over the study period

**Fig. 1:**
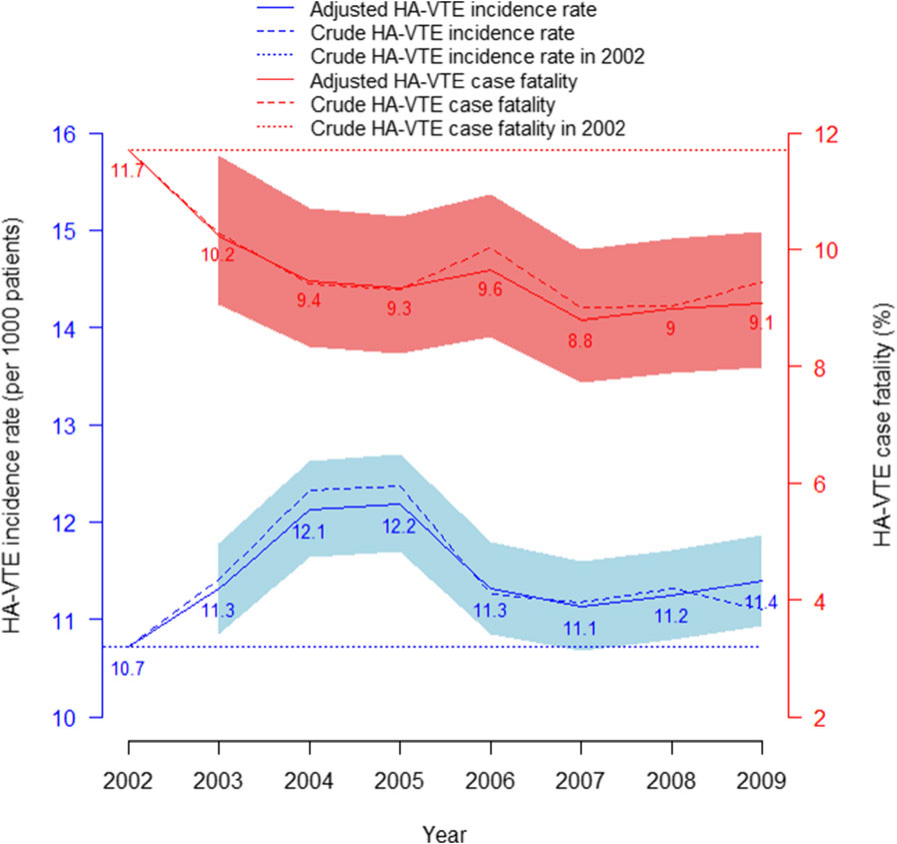
Adjusted trends of HA-VTE (per 1000 patients) and case fatality (%) over the study period

**Fig. 2:**
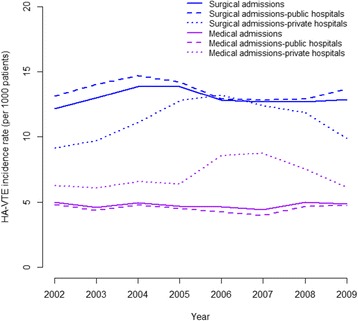
Adjusted trend of HA-VTE stratified by admission and hospital type over the study period

HA-VTE incidence rate increased by 14 % between 2002 and 2004 reaching an adjusted incidence rate of 12.4 per 1000 patients, and then declined during the rest of the study period (Fig. 1). The increase in the HA-VTE incidence rate between 2002 and 2004 occurred in surgical but not medical patients in all hospitals (Fig. 2). However, in private hospitals, the incidence rate of HA-VTE peaked later, with greater increments of 44 and 40 % in 2006 and 2007 for surgical and medical patients, respectively (Fig. 2 and Additional file 4). Higher variations were observed in stratified trends - by public versus private, types of patients, hospital peer groups and surgical types (see Additional file 5).

**Fig. 3:**
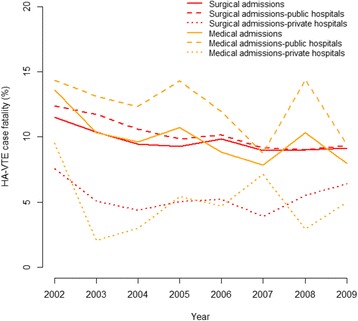
Adjusted trend of post HA-VTE case fatality stratified by admission and hospital type over the study period

The adjusted HA-VTE case fatality rate significantly declined by 22 % from 11.7 to 9.1 % over the study period (Fig. 1). Stratified analysis illustrated in Fig. 3 showed similar declines between public (2009 vs 2002: IRR = 0.74) and private hospitals (2009 vs 2002: IRR = 0.88), surgical (IRR = 0.80) and medical patients (IRR = 0.59). Large principal referral and metropolitan hospitals (coded as A1 and B) exhibited a significant decrease in the HA-VTE associated fatality rate over the study period (see Additional file 5).

### The variation of outcomes between and with hospital peer groups stratified by patients types (medical versus surgical)

Table 2 shows that private hospitals exhibited a larger variation between those hospitals with the lowest, and those with the highest HA-VTE rate, and associated fatality rates within each peer groups compared to public hospitals peer groups. The major private hospital peer group showed the highest variability of HA-VTA between the top and bottom 20 % percentile performers (IRR = 5.33), followed by district group 2 public hospitals (IRR = 2.94) and district private hospital groups (IRR = 2.44). For HA-VTE case fatality, the major private hospital group had the largest variability (IRR = 6.23), followed by the district private hospital group (IRR = 5.72) and then the district group 1 public hospitals (IRR = 3.77). These within group variations were almost replicated for surgical patients, whereas for medical patients, the district group 1 public hospital showed the highest variability for HA-VTE rates (IRR = 4.32) and principal referral public hospital group had the highest variability for HA-VTE case fatality (IRR = 15.65). The significant negative correlations between individual hospital HA-VTE rates and HA-VTEs case fatality rates for private hospital peer groups implied that hospitals with the highest HA-VTE rate tended to have a lower rate of subsequent death. There were no such associations within other public hospitals peer groups.

**Table 2:** Rates, adjusted rate ratios and association of outcomes between the best and worst performers within hospital peer groups

Hospital peer group	Hospital	HA-VTE					HA-VTE case fatality					Correlation coefficient		
	n	Lowest (IR)	Highest (IR)	IRR (95 % CI)			Lowest (%)	Highest (%)	IRR (95 % CI)			(95 % CI)		
All admissions														
Public hospitals														
Principal referral (A1)	17	11.0	22.9	2.09	(2–2.19)	^b^	7.7 %	13.8 %	1.93	(1.64–2.27)	^b^	−0.38	(-0.76–0.19)	
Major metro- & non-metropolitan (B)	22	6.8	12.6	1.70	(1.58–1.84)	^b^	6.9 %	15.0 %	2.13	(1.73–2.63)	^b^	−0.22	(-0.59–0.22)	
District group 1 (C1)	13	5.8	11.7	2.02	(1.77–2.31)	^b^	5.9 %	20.5 %	3.77	(2.41–5.91)	^b^	−0.35	(-0.76–0.25)	
District group 2 (C2)	30	2.8	10.4	2.94	(2.39–3.62)	^b^	4.7 %	14.7 %	3.47	(1.94–6.19)	^b^	0.04	(-0.33–0.39)	
Private hospitals														
Major (21)	10	4.0	24.7	5.33	(4.62–6.16)	^b^	1.4 %	10.5 %	6.23	(4.25–9.13)	^b^	−0.63	(-0.9–0.01)	^a^
District (22)	12	6.0	14.3	2.44	(2.05–2.9)	^b^	2.8 %	12.3 %	5.72	(3.1–10.58)	^b^	−0.82	(-0.95–0.46)	^b^
Medical admissions														
Public hospitals														
Principal referral (A1)	17	3.3	7.6	2.24	(1.75–2.87)	^b^	1.3 %	18.3 %	15.65	(3.73–65.58)	^b^	−0.08	(-0.61–0.49)	
Major metro- & non-metropolitan (B)	22	2.3	4.5	2.03	(1.59–2.58)	^b^	0.0 %	20.1 %				0.29	(-0.15–0.63)	
District group 1 (C1)	13	1.5	5.5	4.32	(2.72–6.86)	^b^	4.0 %	20.0 %	5.81	(1.58–21.41)	^b^	−0.36	(-0.76–0.23)	
District group 2 (C2)	30	2.3	7.0	3.65	(2.64–5.06)	^b^	0.0 %	27.8 %				−0.13	(-0.47–0.24)	
Private hospitals														
Major (21)	10	5.1	13.6	2.52	(1.48–4.29)	^b^	1.1 %	20.0 %				0.32	(-0.39–0.79)	
District (22)	12	3.6	12.9	3.88	(2.55–5.92)	^b^	4.6 %	16.7 %	2.57	(0.66–9.97)		−0.67	(-0.9–0.16)	^a^
Surgical admissions														
Public hospitals														
Principal referral (A1)	17	11.8	25.0	2.12	(2.02–2.22)	^b^	8.9 %	14.0 %	1.74	(1.52–1.99)	^b^	−0.38	(-0.76–0.19)	
Major metro- & non-metropolitan (B)	22	7.9	14.3	1.74	(1.61–1.88)	^b^	7.2 %	14.7 %	2.01	(1.62–2.49)	^b^	−0.21	(-0.58–0.23)	
District group 1 (C1)	13	7.2	13.7	1.88	(1.63–2.16)	^b^	5.8 %	20.8 %	4.11	(2.5–6.75)	^b^	−0.30	(-0.73–0.3)	
District group 2 (C2)	30	2.9	14.7	3.75	(2.83–4.98)	^b^	3.8 %	14.9 %	4.37	(2.06–9.26)	^b^	0.05	(-0.32–0.4)	
Private hospitals														
Major (21)	10	3.6	25.0	6.07	(5.2–7.09)	^b^	1.7 %	11.2 %	5.88	(3.77–9.18)	^b^	−0.36	(-0.81–0.35)	
District (22)	12	5.9	11.8	2.37	(1.99–2.81)	^b^	2.2 %	11.3 %	6.88	(3.24–14.62)	^b^	−0.72	(-0.92–0.25)	^b^

Incidence rates (IR) are crude and reported per 1000 patients

Incidence rate ratios (IRR) and related confident intervals (CI) were obtained using Poisson mixed models and adjusted for patient characteristics. Those hospitals with the lowest rate were set as the reference level

Ungrouped acute peer group within public hospitals was removed from analysis due to small number of hospitals within this group

^a^Significant at 5 %; ^b^significant at 1 %

## Discussion

In this large cohort study of all NSW acute hospitals during 2002–2009, we found that over 1 in 100 patients developed HA-VTE. Of these, one in ten subsequently died during hospitalisation. The adjusted HA-VTE incidence peaked in 2004 and then declined afterwards, while the HA-VTE case fatality decreased by 22 % over the study period. Risk of HA-VTE for surgical patients was double the risk for medical patients, but fatality rate was similar. Medical patients admitted at private hospitals experienced higher risks of HA-VTE; whereas surgical patients were at higher risk of subsequent death in public hospitals. There were significant variations in HA-VTE incidence and case fatality between, and within, hospital peer groups. Among public hospitals, principal referral hospitals had a higher HA-VTE incidence but lower associated deaths compared to the smaller hospitals. Whereas there was no difference between major and smaller private hospitals. Overall, between-hospital variation tended to be higher among private hospitals, particularly for major facilities. Smaller public hospitals often exhibited a larger variation in both rates.

The incidence of HA-VTE in NSW hospitals was almost five fold the rate (11.4 vs 2.4 per 1000 patients with VTE as secondary diagnoses) previously reported in Australia (Australian Institute of Health and Welfare (AIHW) 2010). Our rate among surgical patients was higher than those reported for patients who underwent elective surgery in NSW or U.S. hospitals (13 vs 2–4.5 per 1000 patients) [12, 14, 21]. In contrast, our observed rate for medical patients was broadly consistent with those reported for U.S. hospitals (4.5 vs 2.5 to 5.1 per 1000 patients) [42, 43]. No comparable measures are available for overall HA-VTE case fatality in an Australian setting. For surgical patients, case fatality rate was similar to the previous report in NSW (9.8 % vs. 7.9 and 8.3 %) [14, 21]; for medical patients, it was lower than the U.S hospitals’ rate (9.6 % vs 15 to 16.5 %) [42, 44]. Differences in case identification introduced by varying ICD codes and systems, and inclusion of VTE incidents for all surgical patients in this study, compared to patients who just underwent elective surgery in other reports may have contributed to the discrepancies.

Patients with cancer, vascular and clotting disorders are at high risk of developing VTE [5, 44]. The high fatality rate found among cancer patients is consistent with previous studies reporting cancer as a major risk factor of developing PE and a high fatality rate [45]. Our finding that medical patients were less likely to develop VTE compared to surgical patients echoes the fact that surgery and in particular major procedures such as orthopaedic or AAA repair are known risk factors [1, 21, 46].

The recording and reporting of post-operative VTE as a patient safety outcome may have contributed to halving of the HA-VTE rates amongst surgical patients in the U.S. [12, 15]. No such a drop in Australia was evident [21, 23]. However, the decreasing trajectory in HA-VTE incidents and in particular in associated mortality after adjusting for varied length of stay over time, coincided with the release and adherence with VTE prevention guidelines in Australia [2, 47].

Compliance with VTE prevention guidelines was reportedly suboptimal for hospitalised patients and medical patients were up to 50 % less likely than surgical patients to receive adequate VTE prophylaxis [48–50]. Other studies have shown a smaller improvement in post VTE survival for medical patients [51, 52] which was not evident in our study. Extension of the VTE prevention efforts to medical patients as recently targeted in Australia [11] and elsewhere [53] and routine measurement of associated indictors could reduce incidences and adverse outcome.

We found that the incidence of HA-VTE was lower for medical patients at public versus private hospitals; but public hospitals had higher subsequent case fatalities, in particular for surgical patients. Differences in compliance with VTE prevention guidelines can result in varying VTE measures across hospitals. Altered length of stay have been contributed to the differences in HA-VTE outcomes between two sectors [44, 54], however we here adjusted for length of stay across hospitals, offsetting the influence of shorter stay in private hospitals [30]. A difference in case mix may have contributed to discrepancies between the two sectors.

Our finding that NSW larger hospitals had excess HA-VTE incidents but lower fatalities is consistent with hospital-volume effect reported for HA-VTE [21] and mortality [55–57]. Undertaking patients with higher surgical complexity and multiple comorbidities as well as better diagnosis of high-risk but asymptomatic VTE in larger hospitals [58] may have contributed to elevated HA-VTE rates in major hospitals. On the other hand, underreporting, failure to diagnose or a mis-coding of HA-VTE may be more common amongst smaller hospitals [22, 37, 59]. Subsequently, majority of the identified VTE cases in smaller hospitals are those who developed PE and were more likely to die. A higher rate of diagnosis, and lower case fatality, may reflect a greater adherence to evidence-based treatment guidelines and less failure-to-rescue. Therefore, study of HA-VTE incidents alone may be misleading and more research is required to identify contributing factors to both the incidence rate and case fatality.

Our study has several implications. Expansion of VTE prevention programs to private hospitals and medical patients are required. The large variation of HA-VTE rates between and within different sectors and hospital peer groups suggests that there is room for improvement in the diagnosis, prevention and treatment of HA-VTE. Development of measures for hospital acquired VTE, and the continual monitoring and public reporting of the VTE incidence and mortality for benchmarking and quality improvement purposes at local, regional and national level should be considered [2, 60].

This study benefited from a large population-based cohort across all acute hospitals within the most populated region in Australia. We utilised two measures that were comparable to well-established VTE and case fatality indicators within the patient safety literature, which allowed us to differentiate prevention versus treatment of VTE as well as benchmarking internally and externally. However our study has multiple limitations. The rates of HA-VTE may be prone to two biases: a) overestimation due to possible inclusion of community base VTE cases that were recorded as secondary diagnoses; and b) underestimation due to neglecting VTE cases developed during hospitalisation but not diagnosed prior to discharge [44]. This suggests that bias may be reduced by the utilisation of newly adopted condition onset variables (whether or not morbidity was present on admission) in hospital administrative datasets [61] and data linkage in the follow-up period [62]. The accuracy of our results were limited to the quality of documentation and clinical coding practices [37], and thus can be improved via clinical chart review. Furthermore inclusion of broader confounding factors such as severity of patients such as Charlson and Elixhauser scores based on accurate morbidity data, body mass index and smoking can provide additional insight on the observed trends [63, 64].

## Conclusion

There incidence and case fatality rate of HA-VTE in NSW hospital in-patients is decreasing. The significant variability for HA-VTE incidents and case fatality between medical and surgical patients within public and private sectors and between hospital peer groups suggests potential for further improvement in both prevention and treatment of HA-VTE. Routine measurement and reporting of both HA-VTE incidence and associated mortality can provide policy-makers, clinicians and researchers with opportunities to monitor and improve performance.

## Additional files

Additional file 1: Procedure codes from ICD-10-AM for selected surgical procedures. Procedure codes from ICD-10-AM for selected surgical procedures. (DOCX 13.2 kb) Additional file 2: Patients’ age and severity over the study period. (DOCX 13.8 kb) Additional file 3: Study population, incidence rates and adjusted rate ratios of surgical patients who developed HA-VTE and associated case fatality. (DOCX 29 kb) Additional file 4: Study population, incidence rates and adjusted rate ratios of medical patients who developed HA-VTE and associated case fatality. (DOCX 24 kb) Additional file 5: Annual incidence rates and adjusted rate ratios of patients who developed HA-VTE and associated case fatality. (DOCX 58 kb)

## Abbreviations

AAA: Abdominal aortic aneurysm
APDC: Admitted patient data collection
CABG: Coronary-artery bypass graft
CI: Confidence interval
DVT: Deep venous thrombosis
HA-VTE: Hospital-acquired venous thromboembolism
ICD-10-AM: International Statistical Classification of Diseases and Related Health Problems, Tenth Revision, Australian Modification
IR: Incidence rate
IRR: Incidence rate ratio
LOS: Length of stay
NSW: New South Wales
PE: Pulmonary embolism
SEIFA: Socio-economic indices for areas
VTE: Venous thromboembolism
