# Supplementary material for: Incidences and variations of hospital acquired venous thromboembolism in Australian hospitals: a population-based study
Source: BMC Health Serv Res. 2016 Sep 22;16:511. doi: 10.1186/s12913-016-1766-y (PMC5034410; doi:10.1186/s12913-016-1766-y)
Supplement: Additional file 1: — Procedure codes from ICD-10-AM for selected surgical procedures. Procedure codes from ICD-10-AM for selected surgical procedures. (DOCX 13.2 kb) [file 12913_2016_1766_MOESM1_ESM.docx]

Procedure codes from ICD-10-AM for selected surgical procedures

| **Procedure category** | **Code** | | | |
| --- | --- | --- | --- | --- |
| Abdominal aortic aneurysm | 33112-00  33115-00  33118-00  33121-00 | 33151-00  33157-00  33154-00  33160-00 |  |  |
| Coronary artery bypass graft | 38497-00  38497-01  38497-02  38497-03  38497-04  38497-05  38497-06  38497-07 | 38500-00  38500-01  38500-02  38500-03  38500-04  38503-00 | 38503-01 38503-02  38503-03  38503-04 | 90201-00  90201-01  90201-02  90201-03 |
| Cholecystectomy | 30443-00  30445-00  30446-00  30448-00  30449-00 | 30454-01  30455-00 |  |  |
| Total hip replacement | 49318-00  49319-00  49324-00  49327-00 | 49330-00  49333-00  49345-00 |  |  |
| Total knee replacement | 49518-00  49519-00  49521-00  49521-01  49521-02  49521-03  49524-00  49524-01 | 49527-00 49534-00  49530-00  49530-01  49533-00  49554-00 |  |  |
