# Supplementary material for: Incidences and variations of hospital acquired venous thromboembolism in Australian hospitals: a population-based study
Source: BMC Health Serv Res. 2016 Sep 22;16:511. doi: 10.1186/s12913-016-1766-y (PMC5034410; doi:10.1186/s12913-016-1766-y)
Supplement: Additional file 2: — Patients’ age and severity over the study period. (DOCX 13.8 kb) [file 12913_2016_1766_MOESM2_ESM.docx]

**Patients’ age, severity and length of stay over the study period.**

| **Characteristic** | **Year** | | | | | | | | | | | | | | | | **Total** | |
| --- | --- | --- | --- | --- | --- | --- | --- | --- | --- | --- | --- | --- | --- | --- | --- | --- | --- | --- |
|  | **2002** | | **2003** | | **2004** | | **2005** | | **2006** | | **2007** | | **2008** | | **2009** | |  |  |
| **Age (mean, IQ)** | 55.8 | (40-72) | 55.9 | (40-72) | 56.1 | (40-72) | 56.4 | (40-73) | 57.2 | (41-74) | 57.7 | (42-74) | 58.0 | (43-74) | 58.5 | (44-74) | 55.8 | (41-73) |
| **Charlson index (mean, IQ)** | 0.7 | (0-2) | 0.7 | (0-2) | 0.7 | (0-2) | 0.7 | (0-2) | 0.7 | (0-2) | 0.7 | (0-2) | 0.5 | (0-0) | 0.3 | (0-0) | 0.6 | (0-1) |
