# Supplementary material for: Incidences and variations of hospital acquired venous thromboembolism in Australian hospitals: a population-based study
Source: BMC Health Serv Res. 2016 Sep 22;16:511. doi: 10.1186/s12913-016-1766-y (PMC5034410; doi:10.1186/s12913-016-1766-y)
Supplement: Additional file 3: — Study population, incidence rates and adjusted rate ratios of surgical patients who developed HA-VTE and associated case fatality. (DOCX 29 kb) [file 12913_2016_1766_MOESM3_ESM.docx]

Study population, incidence rates and adjusted rate ratios of surgical patients who developed HA-VTE and associated case fatality.

| **Characteristics** | **Surgical patients** | | **HA-VTE** | | | | | |  | **HA-VTE case fatality** | | | | | |
| --- | --- | --- | --- | --- | --- | --- | --- | --- | --- | --- | --- | --- | --- | --- | --- |
|  | **N** | **(%)** | **n** | **(%)** | **IR** | **IRR** | **(95% CI)** |  |  | **n** | **(%)** | **%** | **IRR** | **(95% CI)** |  |
| **Sex** |  |  |  |  |  |  |  |  |  |  |  |  |  |  |  |
| Female | 1,406,812 | (51.00%) | 18,528 | (52.05%) | 13.2 | 1.00 |  |  |  | 1,615 | (46.53%) | 8.7% | 1.00 |  |  |
| Male | 1,351,823 | (49.00%) | 17,068 | (47.95%) | 12.6 | 0.95 | (0.93-0.97) | ** |  | 1,856 | (53.47%) | 10.9% | 1.23 | (1.14-1.31) | ** |
| **Age** |  |  |  |  |  |  |  |  |  |  |  |  |  |  |  |
| >=75yr & <90 | 918,706 | (33.30%) | 14,509 | (40.76%) | 15.8 | 1.00 |  |  |  | 1,706 | (49.15%) | 11.8% | 1.00 |  |  |
| >=18yr & <35yr | 293,871 | (10.65%) | 1,575 | (4.42%) | 5.4 | 0.59 | (0.56-0.63) | ** |  | 55 | (1.58%) | 3.5% | 0.25 | (0.19-0.32) | ** |
| >=35yr & <55yr | 570,714 | (20.69%) | 5,078 | (14.27%) | 8.9 | 0.88 | (0.85-0.91) | ** |  | 339 | (9.77%) | 6.7% | 0.49 | (0.43-0.55) | ** |
| >=55yr & <75yr | 975,344 | (35.36%) | 14,434 | (40.55%) | 14.8 | 1.11 | (1.09-1.14) | ** |  | 1,371 | (39.50%) | 9.5% | 0.79 | (0.73-0.85) | ** |
| **Marital status** |  |  |  |  |  |  |  |  |  |  |  |  |  |  |  |
| Married | 1,508,046 | (54.67%) | 19,282 | (54.17%) | 12.8 | 1.00 |  |  |  | 1,945 | (56.04%) | 10.1% | 1.00 |  |  |
| Single | 1,126,594 | (40.84%) | 14,805 | (41.59%) | 13.1 | 0.92 | (0.90-0.94) | ** |  | 1,350 | (38.89%) | 9.1% | 0.90 | (0.84-0.97) | ** |
| Unknown | 123,995 | (4.49%) | 1,509 | (4.24%) | 12.2 | 0.86 | (0.82-0.91) | ** |  | 176 | (5.07%) | 11.7% | 1.15 | (0.98-1.34) |  |
| **Country of birth** |  |  |  |  |  |  |  |  |  |  |  |  |  |  |  |
| Australia and New Zealand | 1,969,515 | (71.39%) | 24,859 | (69.84%) | 12.6 | 1.00 |  |  |  | 2,394 | (68.97%) | 9.6% | 1.00 |  |  |
| UK, US & Canada | 191,874 | (6.96%) | 2,677 | (7.52%) | 14.0 | 1.00 | (0.96-1.04) |  |  | 257 | (7.40%) | 9.6% | 0.91 | (0.80-1.04) |  |
| Non-English Europe | 274,482 | (9.95%) | 4,309 | (12.11%) | 15.7 | 0.99 | (0.96-1.02) |  |  | 459 | (13.22%) | 10.7% | 0.97 | (0.87-1.07) |  |
| North Africa | 52,566 | (1.91%) | 465 | (1.31%) | 8.8 | 0.68 | (0.62-0.74) | ** |  | 47 | (1.35%) | 10.1% | 1.12 | (0.83-1.49) |  |
| Asia | 87,926 | (3.19%) | 948 | (2.66%) | 10.8 | 0.77 | (0.72-0.82) | ** |  | 90 | (2.59%) | 9.5% | 1.07 | (0.87-1.32) |  |
| Others | 182,272 | (6.61%) | 2,338 | (6.57%) | 12.8 | 0.94 | (0.90-0.99) | * |  | 224 | (6.45%) | 9.6% | 1.04 | (0.90-1.19) |  |
| **Quartiles of SEIFA** |  |  |  |  |  |  |  |  |  |  |  |  |  |  |  |
| 1st quartile (most disadvantaged) | 712,868 | (25.84%) | 8,673 | (24.37%) | 12.2 | 1.00 |  |  |  | 859 | (24.75%) | 9.9% | 1.00 |  |  |
| 2nd quartile (disadvantaged) | 695,672 | (25.22%) | 8,157 | (22.92%) | 11.7 | 1.01 | (0.97-1.04) |  |  | 825 | (23.77%) | 10.1% | 1.04 | (0.94-1.14) |  |
| 3rd quartile (advantaged) | 705,215 | (25.56%) | 9,092 | (25.54%) | 12.9 | 0.98 | (0.94-1.01) |  |  | 954 | (27.48%) | 10.5% | 1.12 | (1.01-1.24) | * |
| 4th quartile (most advantaged) | 644,880 | (23.38%) | 9,674 | (27.18%) | 15.0 | 1.00 | (0.96-1.03) |  |  | 833 | (24.00%) | 8.6% | 1.11 | (0.98-1.25) |  |
| **Length of stay** |  |  |  |  |  |  |  |  |  |  |  |  |  |  |  |
| 2-4 days | 1,105,897 | (40.09%) | 3,630 | (10.20%) | 3.3 | 1.00 |  |  |  | 449 | (12.94%) | 12.4% | 1.00 |  |  |
| 4-9 days | 921,391 | (33.40%) | 9,714 | (27.29%) | 10.5 | 2.69 | (2.59-2.80) | ** |  | 736 | (21.20%) | 7.6% | 0.72 | (0.64-0.81) | ** |
| Over 10 days | 731,347 | (26.51%) | 22,252 | (62.51%) | 30.4 | 8.50 | (8.20-8.81) | ** |  | 2,286 | (65.86%) | 10.3% | 0.82 | (0.74-0.91) | ** |
| **Major principal diagnostic diseases**† |  |  |  |  |  |  |  |  |  |  |  |  |  |  |  |
| Cardiac arrhythmias | 49,711 | (1.80%) | 583 | (1.64%) | 11.7 | - |  |  |  | 19 | (0.55%) | 3.3% | - |  |  |
| Chronic pulmonary disease | 98,763 | (3.58%) | 757 | (2.13%) | 7.7 | - |  |  |  | 79 | (2.28%) | 10.4% | - |  |  |
| Coagulopathy | 4,336 | (0.16%) | 176 | (0.49%) | 40.6 | - |  |  |  | 15 | (0.43%) | 8.5% | - |  |  |
| Congestive heart failure | 51,944 | (1.88%) | 887 | (2.49%) | 17.1 | - |  |  |  | 115 | (3.31%) | 13.0% | - |  |  |
| Diabetes with chronic complication | 34,964 | (1.27%) | 443 | (1.24%) | 12.7 | - |  |  |  | 56 | (1.61%) | 12.6% | - |  |  |
| Malignancies | 196,462 | (7.12%) | 3,626 | (10.19%) | 18.5 | - |  |  |  | 779 | (22.44%) | 21.5% | - |  |  |
| Metastatic solid tumour | 45,668 | (1.66%) | 1,367 | (3.84%) | 29.9 | - |  |  |  | 334 | (9.62%) | 24.4% | - |  |  |
| Peripheral vascular disease | 25,160 | (0.91%) | 416 | (1.17%) | 16.5 | - |  |  |  | 58 | (1.67%) | 13.9% | - |  |  |
| Pulmonary circulation disorders | 1,291 | (0.05%) | 62 | (0.17%) | 48.0 | - |  |  |  | 9 | (0.26%) | 14.5% | - |  |  |
| Renal disease | 7,929 | (0.29%) | 102 | (0.29%) | 12.9 | - |  |  |  | 14 | (0.40%) | 13.7% | - |  |  |
| Rheumatic disease | 8,722 | (0.32%) | 166 | (0.47%) | 19.0 | - |  |  |  | 9 | (0.26%) | 5.4% | - |  |  |
| **Surgery type** |  |  |  |  |  |  |  |  |  |  |  |  |  |  |  |
| AAA repair | 2,832 | (0.10%) | 61 | (0.17%) | 21.5 | 1.00 |  |  |  | 13 | (0.37%) | 21.3% | 1.00 |  |  |
| CABG | 28,864 | (1.05%) | 206 | (0.58%) | 7.1 | 0.38 | (0.29-0.51) | ** |  | 27 | (0.78%) | 13.1% | 0.76 | (0.39-1.48) |  |
| Cholecystectomy | 44,708 | (1.62%) | 151 | (0.42%) | 3.4 | 0.52 | (0.39-0.70) | ** |  | 17 | (0.49%) | 11.3% | 0.67 | (0.33-1.39) |  |
| Hip replacement | 41,060 | (1.49%) | 740 | (2.08%) | 18.0 | 1.37 | (1.06-1.78) | * |  | 19 | (0.55%) | 2.6% | 0.23 | (0.11-0.46) | ** |
| Knee replacement | 56,069 | (2.03%) | 2,565 | (7.21%) | 45.7 | 3.78 | (2.94-4.86) | ** |  | 7 | (0.20%) | 0.3% | 0.03 | (0.01-0.07) | ** |
| Other | 2,585,102 | (93.71%) | 31,873 | (89.54%) | 12.3 | 1.13 | (0.88-1.44) |  |  | 3,388 | (97.61%) | 10.6% | 0.65 | (0.37-1.11) |  |
| **Hospital type** |  |  |  |  |  |  |  |  |  |  |  |  |  |  |  |
| Public | 2,186,741 | (79.27%) | 28,995 | (81.46%) | 13.3 | 1.00 |  |  |  | 3,148 | (90.69%) | 10.9% | 1.00 |  |  |
| Private | 571,894 | (20.73%) | 6,601 | (18.54%) | 11.5 | 0.91 | (0.75-1.11) |  |  | 323 | (9.31%) | 4.9% | 0.59 | (0.47-0.75) | ** |
| **Peer hospital groups-Public** |  |  |  |  |  |  |  |  |  |  |  |  |  |  |  |
| Principal referral (A1) | 1,150,035 | (52.59%) | 18,270 | (63.01%) | 15.9 | 1.00 |  |  |  | 1,994 | (63.34%) | 10.9% | 1.00 |  |  |
| Ungrouped acute (A3) | 64,803 | (2.96%) | 711 | (2.45%) | 11.0 | 0.67 | (0.47-0.95) | * |  | 120 | (3.81%) | 16.9% | 2.37 | (1.33-4.22) | ** |
| Major metro- & non-metropolitan (B) | 672,027 | (30.73%) | 7,372 | (25.43%) | 11.0 | 0.74 | (0.61-0.89) | ** |  | 755 | (23.98%) | 10.2% | 1.19 | (0.91-1.57) |  |
| District group 1 (C1) | 178,087 | (8.14%) | 1,685 | (5.81%) | 9.5 | 0.64 | (0.51-0.80) | ** |  | 175 | (5.56%) | 10.4% | 1.71 | (1.22-2.41) | ** |
| District group 2 (C2) | 121,789 | (5.57%) | 957 | (3.30%) | 7.9 | 0.44 | (0.36-0.55) | ** |  | 104 | (3.30%) | 10.9% | 2.22 | (1.55-3.16) | ** |
| **Peer hospital groups-Private** |  |  |  |  |  |  |  |  |  |  |  |  |  |  |  |
| Major (21) | 381,812 | (66.76%) | 4,759 | (72.10%) | 12.5 | 1.00 |  |  |  | 206 | (63.78%) | 4.3% | 1.00 |  |  |
| District (22) | 190,082 | (33.24%) | 1,842 | (27.90%) | 9.7 | 1.09 | (0.69-1.72) |  |  | 117 | (36.22%) | 6.4% | 1.04 | (0.56-1.93) |  |
| **Local health district** |  |  |  |  |  |  |  |  |  |  |  |  |  |  |  |
| Metropolitan | 1,989,436 | (72.12%) | 28,288 | (79.47%) | 14.2 | 1.00 |  |  |  | 2,680 | (77.21%) | 9.5% | 1.00 |  |  |
| Rural & Regional NSW | 769,199 | (27.88%) | 7,308 | (20.53%) | 9.5 | 0.67 | (0.57-0.79) | ** |  | 791 | (22.79%) | 10.8% | 1.11 | (0.92-1.34) |  |
| **Total** | 2,758,635 |  | 35,596 |  | 12.9 |  |  |  |  | 3,471 |  | 9.8% |  |  |  |
| 94,954 (3.3%) cases were excluded due to missing items.  Incidence rates (IR) are crude and reported per 1000 patients.  Incidence rate ratios (IRR) and related confident intervals (CI) were obtained using Poisson mixed models and adjusted for patient (age, gender, marital status, country of birth, socio-economic status (SEIFA), length of stay, and surgery type) and hospital (type, peer group, and health district) characteristics.  † No RR is reported since this characteristic has not been included in the Poisson mixed model.  * Significant at 5%; ** significant at 1%. | | | | | | | | | | | | | | | |
