# Supplementary material for: Incidences and variations of hospital acquired venous thromboembolism in Australian hospitals: a population-based study
Source: BMC Health Serv Res. 2016 Sep 22;16:511. doi: 10.1186/s12913-016-1766-y (PMC5034410; doi:10.1186/s12913-016-1766-y)
Supplement: Additional file 5: — Annual incidence rates and adjusted rate ratios of patients who developed HA-VTE and associated case fatality. (DOCX 58 kb) [file 12913_2016_1766_MOESM5_ESM.docx]

Annual incidence rates and adjusted rate ratios of patients who developed HA-VTE and associated case fatality.

| **Characteristic / Outcome** | |  | **Year** | | | | | | | | | | | | | | | | | | | | | | |  | **Total (2002-2009)** | |  |
| --- | --- | --- | --- | --- | --- | --- | --- | --- | --- | --- | --- | --- | --- | --- | --- | --- | --- | --- | --- | --- | --- | --- | --- | --- | --- | --- | --- | --- | --- |
|  |  |  | **2002** | |  | **2003** | |  | **2004** | |  | **2005** | |  | **2006** | |  | **2007** | |  | **2008** | |  | **2009** | |  | **Linear trend** | |  |
| **All admissions** | |  |  |  |  |  |  |  |  |  |  |  |  |  |  |  |  |  |  |  |  |  |  |  |  |  |  |  |  |
| HA-VTE | N (IR) |  | 4327 | (10.7) |  | 4658 | (11.4) |  | 4952 | (12.3) |  | 5020 | (12.4) |  | 4620 | (11.3) |  | 4730 | (11.2) |  | 4965 | (11.3) |  | 4884 | (11.1) |  | 38156 | (11.5) |  |
|  | IRR (95%CI) |  | 1.00 | - |  | 1.06 | (1.01-1.1) | * | 1.13 | (1.09-1.18) | ** | 1.14 | (1.09-1.18) | ** | 1.06 | (1.01-1.1) | * | 1.04 | (1-1.08) |  | 1.05 | (1.01-1.09) | * | 1.06 | (1.02-1.11) | ** | 0.99 | (0.98-0.99) | ** |
| HA-VTE death | N (%) |  | 506 | (11.7%) |  | 479 | (10.3%) |  | 466 | (9.4%) |  | 468 | (9.3%) |  | 463 | (10%) |  | 426 | (9%) |  | 448 | (9%) |  | 460 | (9.4%) |  | 3716 | (9.7%) |  |
|  | IRR (95%CI) |  | 1.00 | - |  | 0.88 | (0.77-0.99) | * | 0.81 | (0.71-0.92) | ** | 0.80 | (0.7-0.91) | ** | 0.83 | (0.73-0.94) | ** | 0.75 | (0.66-0.85) | ** | 0.77 | (0.68-0.87) | ** | 0.78 | (0.68-0.88) | ** | 0.97 | (0.96-0.98) | ** |
|  |  |  |  |  |  |  |  |  |  |  |  |  |  |  |  |  |  |  |  |  |  |  |  |  |  |  |  |  |  |
| **Public hospitals** | |  |  |  |  |  |  |  |  |  |  |  |  |  |  |  |  |  |  |  |  |  |  |  |  |  |  |  |  |
| HA-VTE | N (IR) |  | 3552 | (11.3) |  | 3830 | (12) |  | 4054 | (12.7) |  | 4070 | (12.1) |  | 3800 | (11.1) |  | 3829 | (10.9) |  | 3973 | (11) |  | 4053 | (11.3) |  | 31161 | (11.5) |  |
|  | IRR (95%CI) |  | 1.00 | - |  | 1.06 | (1.01-1.11) | * | 1.11 | (1.06-1.16) | ** | 1.07 | (1.03-1.12) | ** | 0.98 | (0.94-1.03) |  | 0.97 | (0.93-1.01) |  | 0.99 | (0.94-1.03) |  | 1.04 | (1-1.09) |  | 0.98 | (0.98-0.99) | ** |
| HA-VTE death | N (%) |  | 446 | (12.6%) |  | 439 | (11.5%) |  | 426 | (10.5%) |  | 430 | (10.6%) |  | 424 | (11.2%) |  | 390 | (10.2%) |  | 402 | (10.1%) |  | 405 | (10%) |  | 3362 | (10.8%) |  |
|  | IRR (95%CI) |  | 1.00 | - |  | 0.92 | (0.81-1.05) |  | 0.85 | (0.74-0.97) | * | 0.80 | (0.7-0.91) | ** | 0.81 | (0.71-0.93) | ** | 0.72 | (0.63-0.82) | ** | 0.73 | (0.63-0.83) | ** | 0.74 | (0.65-0.85) | ** | 0.95 | (0.94-0.96) | ** |
|  |  |  |  |  |  |  |  |  |  |  |  |  |  |  |  |  |  |  |  |  |  |  |  |  |  |  |  |  |  |
| **Private hospitals** | |  |  |  |  |  |  |  |  |  |  |  |  |  |  |  |  |  |  |  |  |  |  |  |  |  |  |  |  |
| HA-VTE | N (IR) |  | 775 | (8.8) |  | 828 | (9.2) |  | 898 | (11) |  | 954 | (13.6) |  | 822 | (12.2) |  | 901 | (12.5) |  | 992 | (12.6) |  | 831 | (10.4) |  | 7001 | (11.1) |  |
|  | IRR (95%CI) |  | 1.00 | - |  | 1.04 | (0.95-1.15) |  | 1.20 | (1.09-1.32) | ** | 1.40 | (1.27-1.54) | ** | 1.43 | (1.3-1.58) | ** | 1.36 | (1.23-1.5) | ** | 1.31 | (1.19-1.44) | ** | 1.10 | (0.99-1.21) |  | 1.01 | (1-1.02) | * |
| HA-VTE death | N (%) |  | 60 | (7.7%) |  | 40 | (4.8%) |  | 40 | (4.5%) |  | 38 | (4%) |  | 39 | (4.7%) |  | 36 | (4%) |  | 46 | (4.6%) |  | 55 | (6.6%) |  | 354 | (5.0%) |  |
|  | IRR (95%CI) |  | 1.00 | - |  | 0.63 | (0.42-0.93) | * | 0.59 | (0.4-0.89) | * | 0.63 | (0.42-0.95) | * | 0.66 | (0.44-0.99) | * | 0.51 | (0.34-0.78) | ** | 0.70 | (0.48-1.03) |  | 0.88 | (0.6-1.27) |  | 0.98 | (0.94-1.03) |  |
|  |  |  |  |  |  |  |  |  |  |  |  |  |  |  |  |  |  |  |  |  |  |  |  |  |  |  |  |  |  |
| **Surgical admissions** | |  |  |  |  |  |  |  |  |  |  |  |  |  |  |  |  |  |  |  |  |  |  |  |  |  |  |  |  |
| HA-VTE | N (IR) |  | 3922 | (12.2) |  | 4323 | (13) |  | 4608 | (14) |  | 4720 | (14.1) |  | 4330 | (12.7) |  | 4451 | (12.6) |  | 4650 | (12.6) |  | 4590 | (12.3) |  | 35594 | (12.9) |  |
|  | IRR (95%CI) |  | 1.00 | - |  | 1.07 | (1.02-1.12) | ** | 1.14 | (1.09-1.19) | ** | 1.14 | (1.09-1.19) | ** | 1.05 | (1.01-1.1) | * | 1.04 | (1-1.09) |  | 1.04 | (1-1.09) |  | 1.06 | (1.01-1.1) | ** | 0.99 | (0.98-0.99) | ** |
| HA-VTE death | N (%) |  | 451 | (11.5%) |  | 449 | (10.4%) |  | 435 | (9.4%) |  | 434 | (9.2%) |  | 439 | (10.1%) |  | 404 | (9.1%) |  | 420 | (9%) |  | 439 | (9.6%) |  | 3471 | (9.7%) |  |
|  | IRR (95%CI) |  | 1.00 | - |  | 0.90 | (0.79-1.03) |  | 0.82 | (0.72-0.94) | ** | 0.81 | (0.71-0.92) | ** | 0.86 | (0.75-0.98) | * | 0.78 | (0.68-0.89) | ** | 0.78 | (0.68-0.89) | ** | 0.80 | (0.7-0.91) | ** | 0.97 | (0.96-0.99) | ** |
|  |  |  |  |  |  |  |  |  |  |  |  |  |  |  |  |  |  |  |  |  |  |  |  |  |  |  |  |  |  |
| **Medical admissions** | |  |  |  |  |  |  |  |  |  |  |  |  |  |  |  |  |  |  |  |  |  |  |  |  |  |  |  |  |
| HA-VTE | N (IR) |  | 405 | (5) |  | 335 | (4.4) |  | 344 | (4.7) |  | 304 | (4.3) |  | 289 | (4.3) |  | 279 | (4) |  | 315 | (4.6) |  | 294 | (4.4) |  | 2565 | (4.5) |  |
|  | IRR (95%CI) |  | 1.00 | - |  | 0.92 | (0.8-1.06) |  | 1.00 | (0.86-1.15) |  | 0.94 | (0.81-1.09) |  | 0.93 | (0.8-1.09) |  | 0.89 | (0.76-1.04) |  | 1.00 | (0.86-1.16) |  | 0.98 | (0.84-1.14) |  | 0.99 | (0.97-1.01) |  |
| HA-VTE death | N (%) |  | 55 | (13.6%) |  | 30 | (9%) |  | 31 | (9%) |  | 34 | (11.2%) |  | 24 | (8.3%) |  | 22 | (7.9%) |  | 28 | (8.9%) |  | 21 | (7.1%) |  | 245 | (9.6%) |  |
|  | IRR (95%CI) |  | 1.00 | - |  | 0.76 | (0.47-1.22) |  | 0.71 | (0.45-1.13) |  | 0.79 | (0.5-1.24) |  | 0.65 | (0.39-1.07) |  | 0.58 | (0.34-0.97) | * | 0.76 | (0.47-1.23) |  | 0.59 | (0.34-1) | * | 0.94 | (0.89-1) | * |
|  |  |  |  |  |  |  |  |  |  |  |  |  |  |  |  |  |  |  |  |  |  |  |  |  |  |  |  |  |  |
| **Surgical admissions-**  **Public hospitals** | |  |  |  |  |  |  |  |  |  |  |  |  |  |  |  |  |  |  |  |  |  |  |  |  |  |  |  |  |
| HA-VTE | N (IR) |  | 3210 | (13.1) |  | 3550 | (14.1) |  | 3765 | (14.7) |  | 3810 | (14.1) |  | 3560 | (12.7) |  | 3600 | (12.5) |  | 3706 | (12.5) |  | 3796 | (12.7) |  | 28997 | (13.3) |  |
|  | IRR (95%CI) |  | 1.00 | - |  | 1.07 | (1.02-1.12) | ** | 1.12 | (1.07-1.17) | ** | 1.08 | (1.03-1.13) | ** | 0.98 | (0.94-1.03) |  | 0.98 | (0.93-1.02) |  | 0.98 | (0.94-1.03) |  | 1.04 | (0.99-1.09) |  | 0.98 | (0.98-0.99) | ** |
| HA-VTE death | N (%) |  | 397 | (12.4%) |  | 412 | (11.6%) |  | 401 | (10.7%) |  | 400 | (10.5%) |  | 403 | (11.3%) |  | 372 | (10.3%) |  | 376 | (10.1%) |  | 387 | (10.2%) |  | 3148 | (10.9%) |  |
|  | IRR (95%CI) |  | 1.00 | - |  | 0.95 | (0.83-1.09) |  | 0.86 | (0.75-0.98) | * | 0.80 | (0.69-0.91) | ** | 0.82 | (0.72-0.94) | ** | 0.74 | (0.64-0.85) | ** | 0.73 | (0.63-0.84) | ** | 0.76 | (0.66-0.87) | ** | 0.95 | (0.94-0.97) | ** |
|  |  |  |  |  |  |  |  |  |  |  |  |  |  |  |  |  |  |  |  |  |  |  |  |  |  |  |  |  |  |
| **Medical admissions-**  **Public hospitals** | |  |  |  |  |  |  |  |  |  |  |  |  |  |  |  |  |  |  |  |  |  |  |  |  |  |  |  |  |
| HA-VTE | N (IR) |  | 342 | (4.8) |  | 280 | (4.2) |  | 289 | (4.5) |  | 263 | (4) |  | 238 | (3.8) |  | 229 | (3.6) |  | 267 | (4.3) |  | 257 | (4.2) |  | 2165 | (4.2) |  |
|  | IRR (95%CI) |  | 1.00 | - |  | 0.91 | (0.78-1.07) |  | 0.99 | (0.85-1.16) |  | 0.94 | (0.8-1.1) |  | 0.88 | (0.75-1.04) |  | 0.83 | (0.7-0.98) | * | 0.98 | (0.83-1.15) |  | 0.98 | (0.83-1.15) |  | 0.98 | (0.97-1) |  |
| HA-VTE death | N (%) |  | 49 | (14.3%) |  | 27 | (9.6%) |  | 25 | (8.7%) |  | 30 | (11.4%) |  | 21 | (8.8%) |  | 18 | (7.9%) |  | 26 | (9.7%) |  | 18 | (7%) |  | 214 | (9.9%) |  |
|  | IRR (95%CI) |  | 1.00 | - |  | 0.92 | (0.55-1.52) |  | 0.86 | (0.52-1.43) |  | 1.00 | (0.61-1.63) |  | 0.84 | (0.49-1.44) |  | 0.61 | (0.34-1.08) |  | 1.00 | (0.6-1.68) |  | 0.66 | (0.37-1.17) |  | 0.95 | (0.9-1.02) |  |
|  |  |  |  |  |  |  |  |  |  |  |  |  |  |  |  |  |  |  |  |  |  |  |  |  |  |  |  |  |  |
| **Surgical admissions-**  **Private hospitals** | |  |  |  |  |  |  |  |  |  |  |  |  |  |  |  |  |  |  |  |  |  |  |  |  |  |  |  |  |
| HA-VTE | N (IR) |  | 712 | (9.1) |  | 773 | (9.6) |  | 843 | (11.5) |  | 913 | (14.2) |  | 771 | (12.5) |  | 851 | (12.8) |  | 944 | (12.9) |  | 794 | (10.6) |  | 6601 | (11.5) |  |
|  | IRR (95%CI) |  | 1.00 | - |  | 1.06 | (0.96-1.18) |  | 1.22 | (1.1-1.34) | ** | 1.40 | (1.27-1.54) | ** | 1.44 | (1.3-1.6) | ** | 1.36 | (1.23-1.5) | ** | 1.30 | (1.18-1.43) | ** | 1.08 | (0.98-1.2) |  | 1.00 | (0.99-1.01) |  |
| HA-VTE death | N (%) |  | 54 | (7.6%) |  | 37 | (4.8%) |  | 34 | (4%) |  | 34 | (3.7%) |  | 36 | (4.7%) |  | 32 | (3.8%) |  | 44 | (4.7%) |  | 52 | (6.5%) |  | 323 | (4.9%) |  |
|  | IRR (95%CI) |  | 1.00 | - |  | 0.67 | (0.44-1.01) |  | 0.58 | (0.38-0.89) | * | 0.67 | (0.43-1.03) |  | 0.69 | (0.45-1.05) |  | 0.52 | (0.33-0.8) | ** | 0.73 | (0.49-1.09) |  | 0.84 | (0.57-1.24) |  | 0.98 | (0.93-1.03) |  |
|  |  |  |  |  |  |  |  |  |  |  |  |  |  |  |  |  |  |  |  |  |  |  |  |  |  |  |  |  |  |
| **Medical admissions-**  **Private hospitals** | |  |  |  |  |  |  |  |  |  |  |  |  |  |  |  |  |  |  |  |  |  |  |  |  |  |  |  |  |
| HA-VTE | N (IR) |  | 63 | (6.3) |  | 55 | (5.9) |  | 55 | (6.6) |  | 41 | (7) |  | 51 | (9.3) |  | 50 | (9.6) |  | 48 | (8.5) |  | 37 | (6.7) |  | 400 | (7.2) |  |
|  | IRR (95%CI) |  | 1.00 | - |  | 0.97 | (0.68-1.4) |  | 1.05 | (0.73-1.51) |  | 1.03 | (0.69-1.53) |  | 1.37 | (0.94-1.99) |  | 1.40 | (0.96-2.04) |  | 1.21 | (0.82-1.77) |  | 0.98 | (0.65-1.48) |  | 1.02 | (0.97-1.06) |  |
| HA-VTE death | N (%) |  | 6 | (9.5%) |  | 3 | (5.5%) |  | 6 | (10.9%) |  | 4 | (9.8%) |  | 3 | (5.9%) |  | 4 | (8%) |  | 2 | (4.2%) |  | 3 | (8.1%) |  | 31 | (7.8%) |  |
|  | IRR (95%CI) |  | 1.00 | - |  | 0.22 | (0.05-1) |  | 0.31 | (0.08-1.16) |  | 0.57 | (0.14-2.32) |  | 0.49 | (0.12-2.11) |  | 0.75 | (0.19-2.91) |  | 0.31 | (0.05-1.78) |  | 0.53 | (0.1-2.68) |  | 0.97 | (0.81-1.17) |  |
|  |  |  |  |  |  |  |  |  |  |  |  |  |  |  |  |  |  |  |  |  |  |  |  |  |  |  |  |  |  |
| **Public hospital-**  **Principal referral (A1)** | |  |  |  |  |  |  |  |  |  |  |  |  |  |  |  |  |  |  |  |  |  |  |  |  |  |  |  |  |
| HA-VTE | N (IR) |  | 2174 | (14.4) |  | 2404 | (15.7) |  | 2546 | (16.5) |  | 2460 | (15.1) |  | 2300 | (13.7) |  | 2283 | (13.4) |  | 2453 | (13.8) |  | 2514 | (14.1) |  | 19134 | (14.5) |  |
|  | IRR (95%CI) |  | 1.00 | - |  | 1.08 | (1.02-1.14) | * | 1.13 | (1.07-1.2) | ** | 1.05 | (0.99-1.12) |  | 0.96 | (0.91-1.02) |  | 0.94 | (0.89-1) |  | 0.99 | (0.93-1.04) |  | 1.05 | (0.99-1.11) |  | 0.98 | (0.97-0.99) | ** |
| HA-VTE death | N (%) |  | 277 | (12.7%) |  | 279 | (11.6%) |  | 253 | (9.9%) |  | 246 | (10%) |  | 253 | (11%) |  | 261 | (11.4%) |  | 252 | (10.3%) |  | 248 | (9.9%) |  | 2069 | (10.8%) |  |
|  | IRR (95%CI) |  | 1.00 | - |  | 0.91 | (0.77-1.08) |  | 0.78 | (0.66-0.92) | ** | 0.77 | (0.65-0.92) | ** | 0.85 | (0.71-1) |  | 0.87 | (0.74-1.03) |  | 0.80 | (0.67-0.94) | ** | 0.75 | (0.63-0.89) | ** | 0.97 | (0.96-0.99) | ** |
|  |  |  |  |  |  |  |  |  |  |  |  |  |  |  |  |  |  |  |  |  |  |  |  |  |  |  |  |  |  |
| **Public hospital-**  **Ungrouped acute (A3)** | |  |  |  |  |  |  |  |  |  |  |  |  |  |  |  |  |  |  |  |  |  |  |  |  |  |  |  |  |
| HA-VTE | N (IR) |  | 65 | (7.1) |  | 53 | (5.8) |  | 80 | (8.8) |  | 135 | (14.8) |  | 134 | (14.4) |  | 111 | (11.5) |  | 85 | (9.2) |  | 102 | (11.5) |  | 765 | (10.4) |  |
|  | IRR (95%CI) |  | 1.00 | - |  | 0.84 | (0.58-1.2) |  | 1.24 | (0.89-1.72) |  | 2.02 | (1.5-2.72) | ** | 2.07 | (1.54-2.79) | ** | 1.67 | (1.23-2.26) | ** | 1.30 | (0.94-1.8) |  | 1.64 | (1.2-2.24) | ** | 1.06 | (1.03-1.09) | ** |
| HA-VTE death | N (%) |  | 8 | (12.3%) |  | 9 | (17%) |  | 14 | (17.5%) |  | 20 | (14.8%) |  | 27 | (20.1%) |  | 17 | (15.3%) |  | 16 | (18.8%) |  | 21 | (20.6%) |  | 132 | (17.2%) |  |
|  | IRR (95%CI) |  | 1.00 | - |  | 1.29 | (0.48-3.47) |  | 1.25 | (0.52-3.02) |  | 1.02 | (0.44-2.34) |  | 1.41 | (0.63-3.15) |  | 1.00 | (0.42-2.36) |  | 1.41 | (0.59-3.37) |  | 1.39 | (0.61-3.18) |  | 1.03 | (0.94-1.12) |  |
|  |  |  |  |  |  |  |  |  |  |  |  |  |  |  |  |  |  |  |  |  |  |  |  |  |  |  |  |  |  |
| **Public hospital-Major metro & non-metropolitan (B)** | |  |  |  |  |  |  |  |  |  |  |  |  |  |  |  |  |  |  |  |  |  |  |  |  |  |  |  |  |
| HA-VTE | N (IR) |  | 917 | (9.9) |  | 924 | (9.9) |  | 987 | (10.2) |  | 1080 | (10.2) |  | 981 | (9) |  | 1006 | (8.9) |  | 1020 | (8.9) |  | 1046 | (9.1) |  | 7961 | (9.5) |  |
|  | IRR (95%CI) |  | 1.00 | - |  | 0.99 | (0.91-1.09) |  | 1.03 | (0.94-1.12) |  | 1.05 | (0.96-1.15) |  | 0.93 | (0.85-1.02) |  | 0.92 | (0.84-1) |  | 0.93 | (0.85-1.01) |  | 0.98 | (0.9-1.07) |  | 0.98 | (0.97-0.99) | ** |
| HA-VTE death | N (%) |  | 114 | (12.4%) |  | 107 | (11.6%) |  | 108 | (10.9%) |  | 115 | (10.7%) |  | 104 | (10.6%) |  | 81 | (8.1%) |  | 100 | (9.8%) |  | 88 | (8.4%) |  | 817 | (10.3%) |  |
|  | IRR (95%CI) |  | 1.00 | - |  | 0.89 | (0.68-1.16) |  | 0.85 | (0.66-1.11) |  | 0.84 | (0.65-1.09) |  | 0.82 | (0.63-1.07) |  | 0.63 | (0.47-0.84) | ** | 0.75 | (0.57-0.98) | * | 0.65 | (0.49-0.86) | ** | 0.95 | (0.92-0.97) | ** |
|  |  |  |  |  |  |  |  |  |  |  |  |  |  |  |  |  |  |  |  |  |  |  |  |  |  |  |  |  |  |
| **Public hospital-**  **District group 1 (C1)** | |  |  |  |  |  |  |  |  |  |  |  |  |  |  |  |  |  |  |  |  |  |  |  |  |  |  |  |  |
| HA-VTE | N (IR) |  | 236 | (7.4) |  | 261 | (8.4) |  | 247 | (8) |  | 216 | (7.2) |  | 209 | (7.1) |  | 266 | (8.8) |  | 252 | (8.1) |  | 246 | (8.1) |  | 1933 | (7.9) |  |
|  | IRR (95%CI) |  | 1.00 | - |  | 1.12 | (0.94-1.34) |  | 1.07 | (0.89-1.28) |  | 0.98 | (0.81-1.18) |  | 0.95 | (0.79-1.14) |  | 1.16 | (0.97-1.38) |  | 1.07 | (0.89-1.27) |  | 1.10 | (0.92-1.31) |  | 1.00 | (0.98-1.02) |  |
| HA-VTE death | N (%) |  | 30 | (12.7%) |  | 16 | (6.1%) |  | 26 | (10.5%) |  | 24 | (11.1%) |  | 24 | (11.5%) |  | 23 | (8.6%) |  | 23 | (9.1%) |  | 32 | (13%) |  | 198 | (10.2%) |  |
|  | IRR (95%CI) |  | 1.00 | - |  | 0.48 | (0.26-0.88) | * | 0.91 | (0.54-1.54) |  | 0.95 | (0.56-1.63) |  | 0.89 | (0.52-1.53) |  | 0.67 | (0.39-1.16) |  | 0.71 | (0.41-1.23) |  | 0.98 | (0.59-1.63) |  | 1.01 | (0.95-1.07) |  |
|  |  |  |  |  |  |  |  |  |  |  |  |  |  |  |  |  |  |  |  |  |  |  |  |  |  |  |  |  |  |
| **Public hospital-**  **District group 2 (C2)** | |  |  |  |  |  |  |  |  |  |  |  |  |  |  |  |  |  |  |  |  |  |  |  |  |  |  |  |  |
| HA-VTE | N (IR) |  | 160 | (5.1) |  | 188 | (6) |  | 194 | (6.5) |  | 181 | (6.3) |  | 176 | (6.3) |  | 163 | (5.8) |  | 163 | (6) |  | 145 | (5.4) |  | 1370 | (5.9) |  |
|  | IRR (95%CI) |  | 1.00 | - |  | 1.12 | (0.93-1.42) |  | 1.07 | (1.04-1.57) | * | 0.98 | (1.02-1.56) | * | 0.95 | (0.98-1.5) |  | 1.16 | (0.88-1.37) |  | 1.07 | (0.9-1.39) |  | 1.10 | (0.82-1.29) |  | 1.00 | (0.98-1.02) |  |
| HA-VTE death | N (%) |  | 17 | (10.6%) |  | 28 | (14.9%) |  | 25 | (12.9%) |  | 25 | (13.8%) |  | 16 | (9.1%) |  | 8 | (4.9%) |  | 11 | (6.7%) |  | 16 | (11%) |  | 146 | (10.6%) |  |
|  | IRR (95%CI) |  | 1.00 | - |  | 0.48 | (0.77-2.61) |  | 0.91 | (0.57-2) |  | 0.95 | (0.62-2.16) |  | 0.89 | (0.41-1.65) |  | 0.67 | (0.18-0.99) | * | 0.71 | (0.29-1.37) |  | 0.98 | (0.48-1.9) |  | 1.01 | (0.85-0.99) | * |
|  |  |  |  |  |  |  |  |  |  |  |  |  |  |  |  |  |  |  |  |  |  |  |  |  |  |  |  |  |  |
| **Private hospital-**  **Major (21)** | |  |  |  |  |  |  |  |  |  |  |  |  |  |  |  |  |  |  |  |  |  |  |  |  |  |  |  |  |
| HA-VTE | N (IR) |  | 573 | (10.5) |  | 616 | (10.9) |  | 615 | (12) |  | 691 | (14.9) |  | 535 | (12.2) |  | 608 | (12.7) |  | 722 | (13.3) |  | 601 | (10.9) |  | 4961 | (12.1) |  |
|  | IRR (95%CI) |  | 1.00 | - |  | 1.04 | (0.93-1.17) |  | 1.12 | (1-1.25) |  | 1.34 | (1.19-1.49) | ** | 1.31 | (1.16-1.47) | ** | 1.23 | (1.09-1.38) | ** | 1.23 | (1.1-1.37) | ** | 1.02 | (0.91-1.15) |  | 1.00 | (0.99-1.01) |  |
| HA-VTE death | N (%) |  | 33 | (5.8%) |  | 25 | (4.1%) |  | 22 | (3.6%) |  | 23 | (3.3%) |  | 24 | (4.5%) |  | 23 | (3.8%) |  | 28 | (3.9%) |  | 35 | (5.8%) |  | 213 | (4.3%) |  |
|  | IRR (95%CI) |  | 1.00 | - |  | 0.76 | (0.46-1.28) |  | 0.75 | (0.43-1.28) |  | 0.64 | (0.38-1.1) |  | 0.67 | (0.39-1.13) |  | 0.64 | (0.37-1.09) |  | 0.68 | (0.41-1.13) |  | 1.06 | (0.66-1.72) |  | 1.00 | (0.94-1.06) |  |
|  |  |  |  |  |  |  |  |  |  |  |  |  |  |  |  |  |  |  |  |  |  |  |  |  |  |  |  |  |  |
| **Private hospital-**  **District (22)** | |  |  |  |  |  |  |  |  |  |  |  |  |  |  |  |  |  |  |  |  |  |  |  |  |  |  |  |  |
| HA-VTE | N (IR) |  | 202 | (6.1) |  | 212 | (6.4) |  | 283 | (9.4) |  | 263 | (11.1) |  | 287 | (12.2) |  | 293 | (12.1) |  | 270 | (10.9) |  | 230 | (9.1) |  | 2040 | (9.4) |  |
|  | IRR (95%CI) |  | 1.00 | - |  | 1.04 | (0.86-1.26) |  | 1.44 | (1.2-1.73) | ** | 1.59 | (1.32-1.92) | ** | 1.80 | (1.5-2.16) | ** | 1.76 | (1.46-2.11) | ** | 1.58 | (1.31-1.91) | ** | 1.32 | (1.09-1.6) | ** | 1.04 | (1.02-1.07) | ** |
| HA-VTE death | N (%) |  | 27 | (13.4%) |  | 15 | (7.1%) |  | 18 | (6.4%) |  | 15 | (5.7%) |  | 15 | (5.2%) |  | 13 | (4.4%) |  | 18 | (6.7%) |  | 20 | (8.7%) |  | 141 | (6.9%) |  |
|  | IRR (95%CI) |  | 1.00 | - |  | 0.48 | (0.24-0.86) | * | 0.91 | (0.23-0.77) | ** | 0.95 | (0.24-0.91) | * | 0.89 | (0.25-0.93) | * | 0.67 | (0.18-0.72) | ** | 0.71 | (0.31-1.05) |  | 0.98 | (0.38-1.28) |  | 1.01 | (0.89-1.05) |  |
|  |  |  |  |  |  |  |  |  |  |  |  |  |  |  |  |  |  |  |  |  |  |  |  |  |  |  |  |  |  |
| **Surgical admissions-**  **AAA repair** | |  |  |  |  |  |  |  |  |  |  |  |  |  |  |  |  |  |  |  |  |  |  |  |  |  |  |  |  |
| HA-VTE | N (IR) |  | 12 | (26.3) |  | 9 | (20.2) |  | 10 | (23.1) |  | 3 | (8.2) |  | 6 | (17.8) |  | 7 | (23.6) |  | 8 | (28.9) |  | 6 | (27) |  | 61 | (21.5) |  |
|  | IRR (95%CI) |  | 1.00 | - |  | 0.80 | (0.33-1.93) |  | 0.94 | (0.4-2.2) |  | 0.35 | (0.1-1.27) |  | 0.72 | (0.27-1.94) |  | 0.89 | (0.34-2.29) |  | 1.16 | (0.47-2.89) |  | 1.06 | (0.39-2.87) |  | 1.01 | (0.9-1.14) |  |
|  |  |  |  |  |  |  |  |  |  |  |  |  |  |  |  |  |  |  |  |  |  |  |  |  |  |  |  |  |  |
| **Surgical admissions-CABG** | |  |  |  |  |  |  |  |  |  |  |  |  |  |  |  |  |  |  |  |  |  |  |  |  |  |  |  |  |
| HA-VTE | N (IR) |  | 24 | (5.5) |  | 23 | (5.4) |  | 31 | (8.3) |  | 21 | (6.1) |  | 25 | (7.3) |  | 25 | (7.3) |  | 30 | (9) |  | 27 | (9.2) |  | 206 | (7.1) |  |
|  | IRR (95%CI) |  | 1.00 | - |  | 1.01 | (0.57-1.79) |  | 1.39 | (0.82-2.38) |  | 1.00 | (0.56-1.8) |  | 1.21 | (0.69-2.13) |  | 1.26 | (0.72-2.21) |  | 1.50 | (0.87-2.57) |  | 1.51 | (0.87-2.63) |  | 1.07 | (1.01-1.14) | * |
|  |  |  |  |  |  |  |  |  |  |  |  |  |  |  |  |  |  |  |  |  |  |  |  |  |  |  |  |  |  |
| **Surgical admissions-** **Cholecystectomy** | |  |  |  |  |  |  |  |  |  |  |  |  |  |  |  |  |  |  |  |  |  |  |  |  |  |  |  |  |
| HA-VTE | N (IR) |  | 16 | (2.5) |  | 16 | (2.6) |  | 25 | (4.3) |  | 27 | (4.9) |  | 15 | (2.8) |  | 15 | (2.8) |  | 19 | (3.7) |  | 18 | (3.6) |  | 151 | (3.4) |  |
|  | IRR (95%CI) |  | 1.00 | - |  | 1.05 | (0.52-2.13) |  | 1.68 | (0.89-3.19) |  | 1.76 | (0.93-3.31) |  | 1.13 | (0.55-2.31) |  | 1.06 | (0.51-2.17) |  | 1.48 | (0.75-2.93) |  | 1.46 | (0.73-2.91) |  | 1.02 | (0.95-1.1) |  |
|  |  |  |  |  |  |  |  |  |  |  |  |  |  |  |  |  |  |  |  |  |  |  |  |  |  |  |  |  |  |
| **Surgical admissions-**  **Hip replacement** | |  |  |  |  |  |  |  |  |  |  |  |  |  |  |  |  |  |  |  |  |  |  |  |  |  |  |  |  |
| HA-VTE | N (IR) |  | 72 | (15.3) |  | 100 | (20.6) |  | 103 | (21.4) |  | 110 | (21.6) |  | 92 | (19.6) |  | 87 | (17.5) |  | 99 | (17) |  | 77 | (12.6) |  | 740 | (18.0) |  |
|  | IRR (95%CI) |  | 1.00 | - |  | 1.40 | (1.04-1.89) | * | 1.57 | (1.16-2.12) | ** | 1.74 | (1.29-2.34) | ** | 1.86 | (1.37-2.54) | ** | 1.61 | (1.18-2.2) | ** | 1.48 | (1.09-2.01) | * | 1.16 | (0.84-1.6) |  | 0.98 | (0.95-1.02) |  |
|  |  |  |  |  |  |  |  |  |  |  |  |  |  |  |  |  |  |  |  |  |  |  |  |  |  |  |  |  |  |
| **Surgical admissions-**  **Knee replacement** | |  |  |  |  |  |  |  |  |  |  |  |  |  |  |  |  |  |  |  |  |  |  |  |  |  |  |  |  |
| HA-VTE | N (IR) |  | 276 | (48.8) |  | 316 | (54.2) |  | 353 | (58.5) |  | 421 | (58.3) |  | 277 | (39) |  | 324 | (43.7) |  | 341 | (41.1) |  | 257 | (30.2) |  | 2565 | (45.8) |  |
|  | IRR (95%CI) |  | 1.00 | - |  | 1.21 | (1.03-1.43) | * | 1.34 | (1.14-1.57) | ** | 1.45 | (1.24-1.69) | ** | 1.30 | (1.09-1.54) | ** | 1.30 | (1.11-1.53) | ** | 1.10 | (0.94-1.29) |  | 0.86 | (0.72-1.02) |  | 0.94 | (0.93-0.96) | ** |
|  |  |  |  |  |  |  |  |  |  |  |  |  |  |  |  |  |  |  |  |  |  |  |  |  |  |  |  |  |  |
| **Surgical admissions-**  **Other** | |  |  |  |  |  |  |  |  |  |  |  |  |  |  |  |  |  |  |  |  |  |  |  |  |  |  |  |  |
| HA-VTE | N (IR) |  | 3522 | (11.7) |  | 3859 | (12.4) |  | 4086 | (13.3) |  | 4140 | (13.2) |  | 3920 | (12.2) |  | 3993 | (12) |  | 4153 | (12) |  | 4205 | (12) |  | 31878 | (12.3) |  |
|  | IRR (95%CI) |  | 1.00 | - |  | 1.21 | (1.01-1.1) | * | 1.34 | (1.06-1.16) | ** | 1.45 | (1.06-1.16) | ** | 1.30 | (0.98-1.08) |  | 1.30 | (0.97-1.06) |  | 1.10 | (0.98-1.07) |  | 0.86 | (1.02-1.12) | ** | 0.94 | (0.98-0.99) | ** |
| HA-VTE death | N (%) |  | 440 | (12.5%) |  | 443 | (11.5%) |  | 427 | (10.5%) |  | 424 | (10.2%) |  | 428 | (10.9%) |  | 394 | (9.9%) |  | 402 | (9.7%) |  | 430 | (10.2%) |  | 3388 | (10.6%) |  |
|  | IRR (95%CI) |  | 1.00 | - |  | 0.91 | (0.8-1.04) |  | 0.83 | (0.73-0.95) | ** | 0.81 | (0.71-0.92) | ** | 0.85 | (0.75-0.98) | * | 0.78 | (0.68-0.89) | ** | 0.77 | (0.67-0.88) | ** | 0.80 | (0.7-0.91) | ** | 0.97 | (0.96-0.98) | ** |
| Incidence rates (IR) are crude and reported per 1000 patients.  Incidence rate ratios (IRR) and related confident intervals (CI) were obtained using Poisson mixed models and appropriately adjusted for patient (age, gender, marital status, country of birth, socio-economic status (SEIFA), length of stay, and surgery type) and hospital (type, peer group, and health district) characteristics.    Year 2002 was set as the reference level in models with discrete years.  Annual HA-VTE case fatality rates were not investigated for surgery types, except “Other”, due to very low incidence rates.  * Significant at 5%; ** significant at 1%. | | | | | | | | | | | | | | | | | | | | | | | | | | | | | |
